# Rapid Detection of SARS-CoV‑2 Spike Protein Using a Fully 3D-Printed Electrochemical Biosensor

**DOI:** 10.1021/acsomega.5c09067

**Published:** 2025-12-11

**Authors:** Dayenny L. D’Amato, Natália M. Caldas, Lucas V. de Faria, Ana Beatriz C. Souza, Guilherme P. Oliveira, Diego O. Costa, Mikaelly O. B. de Sousa, Rafael M. Dornellas, Célia M. Ronconi

**Affiliations:** † Departamento de Química Inorgânica, Universidade Federal Fluminense, Campus Do Valonguinho, Outeiro São João Batista S/N, Centro, Niterói, RJ 24020-150, Brazil; ‡ Departamento de Química Analítica, Universidade Federal Fluminense, Campus Do Valonguinho, Outeiro São João Batista S/N, Centro, Niterói, RJ 24020-150, Brazil; § Departamento de Química Analítica, 28125 Universidade Federal Do Rio de Janeiro, Campus Fundão, Avenida Athos da Silveira Ramos 149, Cidade Universitária, Rio de Janeiro, RJ 21941-590, Brazil

## Abstract

3D printing has been used for the rapid and low-cost fabrication of sensors across various fields. Multimaterial additive manufacturing enables the simultaneous deposition of conductive and structural materials, allowing the development of integrated electrochemical biosensors without the need for postprocessing. In this work, we report a 3-in-1 3D-printed electrochemical biosensor for the rapid detection of the SARS-CoV-2 spike protein (S Ptn). The biosensor consists of a graphite/polylactic acid (G/PLA) working electrode modified with gold nanoparticles conjugated to polyclonal antibodies (pAb–AuNPs), and carbon black/PLA-based reference and auxiliary electrodes. Detection is achieved via an indirect immunoassay, measuring the decrease in cathodic current of a [Fe­(CN)_6_]^3–/4–^ redox probe using cyclic voltammetry. The biosensor exhibited a detection limit of 0.76 pM, a linear range from 2.5 to 10 μg L^–1^, and a total assay time of under 5 min. Selectivity was confirmed against other viral proteins, including those from Dengue, Zika, and Chikungunya viruses. Tests in human serum demonstrated the sensor’s robustness and applicability for complex matrices. These results highlight the potential of 3D-printed multimaterial biosensors for rapid and selective viral diagnostics.

## Graphical abstract

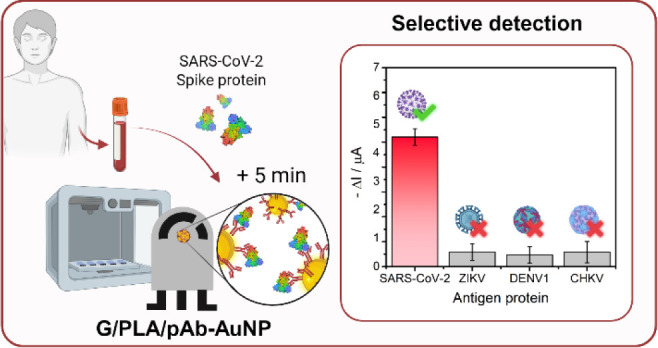

## Introduction

The COVID-19 pandemic has emphasized the global need for rapid, sensitive, and low-cost diagnostic technologies, particularly for point-of-care applications.^1–6^ Among various platforms, electrochemical biosensors have attracted attention due to their speed, miniaturization potential, and compatibility with complex biological matrices.^7–10^

In parallel, 3D printing technologies have emerged as powerful tools for analytical devices with high spatial resolution and geometric flexibility.^11,12^ In particular, multimaterial additive manufacturing enables the simultaneous deposition of conductive and structural components, allowing for the direct fabrication of integrated, ready-to-use electrochemical sensors without postprocessing or assembly.^13^ This combined with biorecognition molecules for detecting specific analytes, new compact, 3-in-1 ready-to-use portable devices can be developed, operating with low volume and enhanced signal.^13,14^ These biosensors rely on the specific interaction between a biorecognition element, such as an antibody, aptamer, or genetic sequences, and a viral marker, which can be measured through a change in an electrochemical signal.^15^

Several reports have explored 3D-printed sensors for viral detection, including SARS-CoV-2, targeting antibodies, nucleic acids and proteins.^16–23^ The pioneering contribution of Muñoz and Pumera^24^ first demonstrated the use of 3D-printed electrodes for spike protein (S Ptn) detection through an indirect impedimetric competitive immunoassay, reaching a limit of detection of 1.1 nM. Importantly, this study established the concept that additive manufacturing could provide customizable biosensing platforms adaptable to different antigen–antibody systems. Building on this concept, Silva et al.^25^ exploited the carboxyl groups of polylactic acid in 3D-printed carbon black/polylactic acid (CB/PLA) electrodes to covalently immobilize anti-S Ptn antibodies via the EDC/NHS method. By directly anchoring the antibodies to the electrode surface, this approach eliminated the need for metallic nanoparticles, thereby offering a cost-effective alternative for biosensor fabrication. However, despite this simplification, the method still required incubation times of 60 min, which represents a major drawback for rapid point-of-care testing (PoCT). In another study,^26^ they focus on nucleic acid detection, employing a gold-modified 3D-printed graphene electrode to target cDNA from SARS-CoV-2. Along a similar line, Crevillen et al.^27^ reported a 3D-printed genosensor integrated into a microfluidic chip for direct RNA detection. While both nucleic acid-based devices offered promising sensitivity and miniaturization, they still relied on prior amplification steps like PCR, which prolong the analysis and hinder true rapid diagnostics. Taken together, these studies clearly establish the versatility of 3D-printed electrodes for biosensing, demonstrating applications that span from antigen detection to nucleic acid recognition. Yet, despite these advances, they share common limitations: either long incubation times or dependence on nucleic acid amplification. These factors restrict their applicability in fast, low-cost, point-of-care diagnostics.

In this work, we address these limitations by developing a compact, fully 3D-printed, multimaterial electrochemical biosensor for the detection of the SARS-CoV-2 S Ptn (Scheme 1). Our device integrates the working, reference, and counter electrodes within a single printed platform, ensuring reproducibility and portability. Detection is achieved through an indirect immunoassay, in which gold nanoparticle–antibody conjugates are immobilized onto the graphite/polylactic acid (G/PLA) working electrode through a one-step modification, overcoming the multiple functionalization steps commonly observed in previous works. The biosensing mechanism relies on monitoring the decrease in cathodic current of the [Fe­(CN)_6_]^3–/4–^ redox couple by cyclic voltammetry upon antigen binding. This design significantly reduces assay time compared to previous antibody-immobilization strategies and avoids nucleic acid amplification steps, while maintaining picomolar-level sensitivity. The biosensor also achieves high selectivity against other viral proteins (Zika, Dengue, Chikungunya) and reliable performance in human serum samples, indicating its suitability for real diagnostic conditions.

**Scheme 1:**
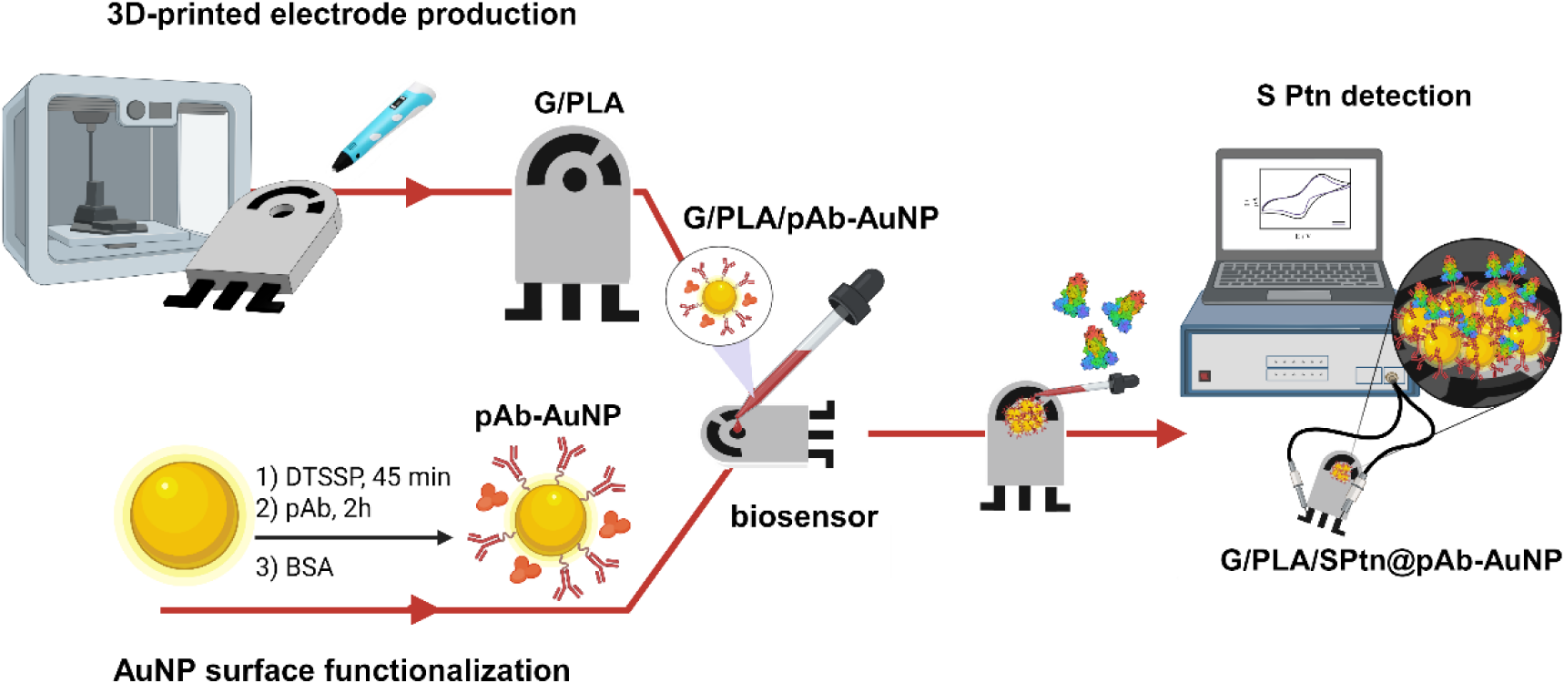
Graphical Representation of the 3D-Printed Biosensor Preparation^a^

a Created with biorender.com.

## Experimental Section

### Materials and Methods

All reagents were used as received without further purification. Gold­(III) chloride trihydrate (HAuCl_4_·3H_2_O, 99.995%), sodium citrate dihydrate (Na_3_C_6_H_5_O_7_·2H_2_O, 99%), 3,3′-dithiobis­(sulfosuccinimidyl propionate) (DTSSP), bovine serum albumin (BSA, 96%), graphite (G) powder (20 μm, 98%) and human serum were purchased from Sigma-Aldrich (St. Louis, USA). Chloroform (99.8%) was acquired at Synth (Diadema, Brazil). Potassium chloride (KCl, 99.5%) was purchased from Merck (Darmstadt, Germany), while potassium ferricyanide (K_3_[Fe­(CN)_6_], 99.0% wt.) and potassium ferrocyanide (K_4_[Fe­(CN)_6_], 99.0% wt.) were obtained from Vetec (Rio de Janeiro, Brazil). For the electrochemical measurements involving the [Fe­(CN)_6_]^3–/4–^, redox pair, with 0.1 mol L^–1^ KCl solution was used as the supporting electrolyte. The electrode fabrication process employed PETG filament from GTMax (São Paulo, Brazil), conductive filament from Protopasta (Washington, USA), and PLA pellets obtained from 3DLAB (Minas Gerais, Brazil).

The anti-S Ptn antibody was obtained from hyperimmune equine serum produced by Instituto Vital Brazil, following immunization with recombinant SARS-CoV-2 S Ptn. The S Ptn antigen was produced by the Cell Culture Engineering Laboratory (LECC) at the Federal University of Rio de Janeiro (UFRJ).^28^ The envelope (E2) protein from Chikungunya virus and the nonstructural protein 1 (NS1) from Dengue virus serotype 1 and Zika virus were purchased from Rheabiotech (Campinas, Brazil).

The bioconjugated pAb–AuNP synthesis was carried out under continuous mixing using an orbital shaker (IKA KS 4000ic control, Staufen, Germany), while purification and concentration steps were performed with a microcentrifuge (MIKRO 220 R, Hettich, Tuttlingen, Germany).

Cyclic voltammetry (CV) measurements were performed using an Ivium CompactStat Technologies (Eindhoven, Netherlands) potentiostat/galvanostat using IviumSoft software interface, using a 5 mV step potential and a 50 mV s^–1^ scan rate in the potential range from −0.6 to +0.6 V.

Fourier-transform infrared spectroscopy (FTIR) spectra were recorded using a Nicolet iS50 spectrophotometer (Thermo Scientific, Waltham, USA) equipped with an ATR accessory (diamond crystal). Measurements were acquired in the range of 4000 to 600 cm^–1^ in transmittance mode, with a resolution of 4 cm^–1^ and 32 scans per spectrum at room temperature.

Zeta potential measurements were obtained in a Zetasizer Advance Lab Blue instrument from Malvern (Great Malvern, England) at 25 °C in a DTS1070 cuvette.

X-ray powder diffraction (XRD) patterns were collected using a Rigaku MiniFelx II diffractometer with a Cu Kα radiation (λ = 1.5406 Å), operating at 30 kV and 15 mA. Data were acquired over a 2θ range of 5–80°, with a step size of 0.05° and a scan rate of 2°/min. Scanning Electron Microscopy (SEM) and Energy Dispersive X-ray Spectroscopy (EDS) were performed in a FEI Magellan 400 equipped with a field emission gun (FEG) (Hillsboro, USA). SEM images were acquired at accelerating voltage of 2 kV with an 0.20 nA current. For the EDS mapping and spectra, a 5 kV voltage and a 6.4 nA current were used.

Contact angle measurements were performed to qualitatively evaluate the wettability changes on the electrode’s surfaces. The measurements were conducted using a setup in which a pipet was maintained perpendicular (90°) to the electrode using a retort stand and a clamp, and a smartphone camera was aligned with the electrode surface. A 20 μL drop of deionized water was placed on the electrode surface, and photographs were captured 10 s after deposition using an Iphone 13. The contact angles were estimated from the droplet images using the ImageJ software. All measurements were performed at room temperature.

### Preparation of the G/PLA Composite Material

The G/PLA composite (40:60 wt.) was prepared according to a procedure reported previously with minor modifications.^29^ PLA was initially dissolved in approximately 150 mL of an acetone/chloroform mixture (3:1 v/v) at 70 °C under continuous stirring. Graphite, used as the conductive filler, was then added to the PLA solution until reaching the desired ratio of 40:60 wt. The mixtures were kept at 70 °C under stirring for 1 h. Subsequently, the composite was precipitated in ethanol, filtered, and dried in an oven at 70 °C for 24 h. The resulting solid was cut into 1 cm pieces and extruded using a Filmaq 3D extruder (Curitiba, Brazil) set at 195 °C and maximum motor rotation, yielding filaments with a diameter of 1.75 mm, which were stored in plastic bags protected from moisture at room temperature.

### Fabrication of 3-in-1 3D-Printed Electrodes

The working electrodes were designed as shown in Figure 1 using Solidworks 3D CAD software in combination with the Bambulab Studio slicer (220 °C printed temperature, 12 mm layer height, 90° infill direction, 5 top shells, 5 wall loops, and printing speed between 50 mm s^–1^ and 150 mm s^–1^). PETG filament was initially printed on a Bambulab A1 mini printer (Shenzhen, China), equipped with an AMS Lite multimaterial system, to fabricate the nonconductive parts of the electrodes. Conductive Protopasta filament was employed to fabricate the carbon black/PLA pseudoreference electrode (CB/PLA electrode, 14.2 mm^2^ area) and the auxiliary electrode (45.7 mm^2^ area), as well as the electrical contacts of these electrodes and the working electrode. A cylindrical cavity (1.8 mm depth and 5.5 mm diameter) at the conductive end of the working electrode was filled with the conductive G/PLA composite filament (40:60 wt.) using a Sanmersen 3D pen (Shenzhen, China). Finally, the electrode surfaces were subjected to a simple mechanical pretreatment with 600- and 1200-grit sandpapers to remove any residual material. The Supporting Information includes a detailed technical drawing of the electrode and the photograph of the electrodes (Figure S1).

**Figure 1:**
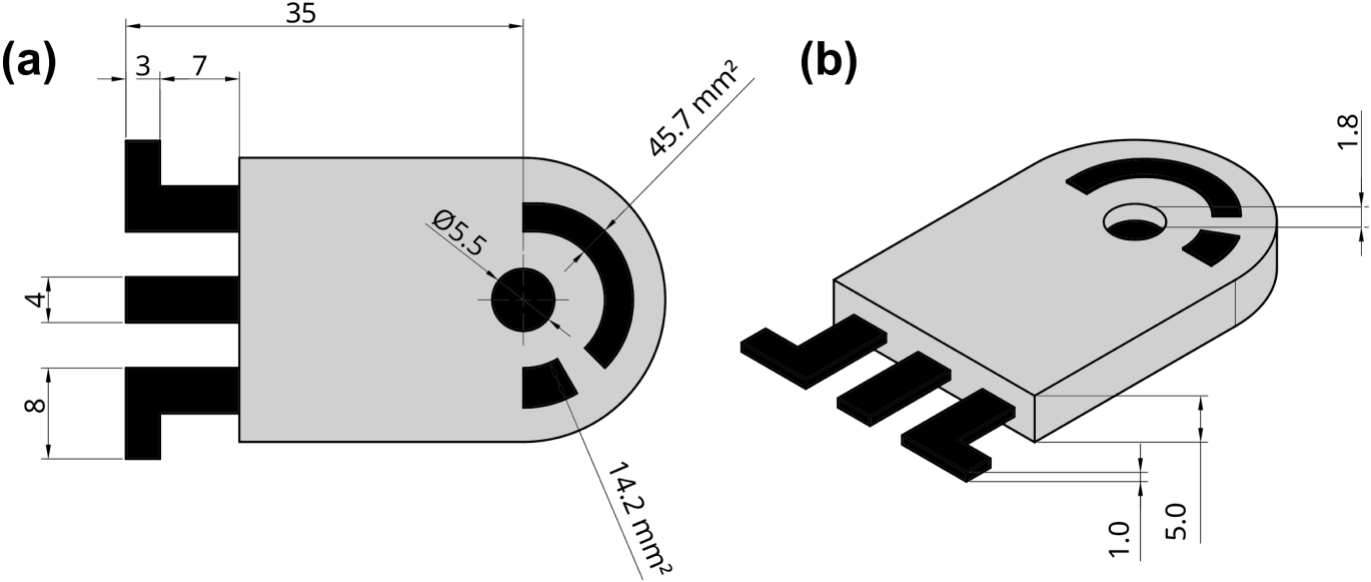
Top (a) and side (b) views with detailed measurements of the 3-in-1 3D-printed electrode developed for the manufacture of electrochemical biosensors.

### Bioconjugate Preparation

Polyclonal anti-SARS-CoV-2 spike protein antibodies were covalently conjugated to 130 nm gold nanoparticles (AuNPs) using the cross-linker 3,3′-dithiobis­(sulfosuccinimidyl propionate) (DTSSP), following a previously reported procedure.^6,30^ Briefly, 400 μL of the AuNP dispersion was washed with 800 μL of deionized water and centrifuged at 3000 rpm for 4 min at 25 °C. After removing 1.0 mL of the supernatant, the AuNPs were redispersed in 948 μL of deionized water. The pH was adjusted to 8.9 by adding 50 μL of borate buffer solution (50 mM), followed by 2 μL of DTSSP solution (0.1 mM). The dispersion was stirred at 300 rpm for 45 min at 25 °C.

After the functionalization, the nanoparticles were washed (3×) with 2 mM borate buffer. Then, 12 μL of polyclonal anti-S Ptn antibodies (1.0 mg mL^–1^) were added, and the mixture was incubated under stirring (300 rpm, 25 °C) for 2 h. Finally, the bioconjugate was washed (2×) with filtered 2 mM borate buffer containing 0.25% (w/v) BSA, yielding the final product, referred to as pAb–AuNP.

### Electrode Surface Functionalization

The working electrode was functionalized via the drop-casting method. The previously prepared dispersion was concentrated at a 1:4 ratio using centrifugation, resulting in a final Au concentration of 1 mg mL^–1^. Subsequently, 20 μL of the concentrated pAb–AuNP was drop-cast onto the electrode surface using a micropipette. The electrode was then left to dry at room temperature for up to 2 h, yielding the final biosensor named G/PLA/pAb–AuNP.

### Protein Recognition Tests

Time-dependent immunoassays were conducted to evaluate the interaction kinetics between the SARS-CoV-2 S Ptn and the fabricated G/PLA/pAb–AuNP biosensor. Cyclic voltammetry (CV) measurements were performed after varying incubation times ranging from 1 to 30 min. For these tests, a 5 μg L^–1^ dispersion of S Ptn was prepared in 0.1 M phosphate buffer (pH 7.0) containing 1 mM potassium ferrocyanide, and 400 μL of this dispersion was applied directly onto the 3-in-1 3D-printed electrodes.

To determine the limit of detection (LOD) and the linear detection range, CV measurements were carried out using S Ptn dispersion at concentrations ranging from 2.5 to 30 μg L^–1^ under identical electrolyte conditions. An incubation time of 5 min was used for all concentration-dependent assays. All experiments were performed at room temperature and repeated in triplicate to ensure reproducibility. The resulting voltammograms were analyzed by monitoring changes in the cathodic peak current as a function of S Ptn binding. Control experiments were conducted under the same conditions using phosphate buffer (0.1 M, pH 7.0) with 1 mM ferrocyanide, but without S Ptn.

To assess the selectivity of the G/PLA/pAb–AuNP biosensor, the same procedure was applied using 10 pM solutions of unrelated viral proteins, including the envelope (E2) protein from Chikungunya virus (CHKV) and the nonstructural protein 1 (NS1) from Zika (ZIKV) and Dengue type 1 (DENV1) viruses.

### Applicability in Human Serum

To evaluate the accuracy and applicability of the method in complex biological matrices, human serum samples (diluted 1:10) were analyzed using the standard addition method. Before the electrochemical measurements, the samples were spiked with S Ptn at two concentration levels (2.5 and 3.5 μg L^–1^).

## Results and Discussion

### Electrodes Characterization

Scanning electron microscopy (SEM) was performed to investigate the surface morphology of the G/PLA, G/PLA/AuNP, and the G/PLA/pAb–AuNP electrodes (Figure 2a–c). The images of the electrodes showed the graphite layers. For the electrodes modified with AuNPs, the nanoparticles were clearly observed on their surfaces. The AuNPs exhibited an aggregated distribution on the G/PLA/AuNP electrode (Figure 2b), whereas in the G/PLA/pAb–AuNP electrode they were uniformly dispersed (Figure 2c). This difference can be attributed to the presence of antibodies conjugated to the nanoparticles, which modify the surface charge and provide steric hindrance that prevents aggregation during the drying process. Zeta-potential measurements confirmed a shift from −29.2 ± 1.9 mV for citrate-stabilized AuNPs to −21.0 ± 1.3 mV for pAb–AuNP, indicating partial neutralization of the negative charge after antibody binding. Although the reduction in surface charge could lower electrostatic repulsion, the protein layer surrounding the nanoparticles prevents metal–metal contact. As a result, the pAb–AuNPs form a more uniform layer on the electrode surface, maximizing the effective binding area for antigen recognition and improving both sensitivity and overall device performance. Additional SEM images are shown in Support Information (Figure S2).

**Figure 2:**
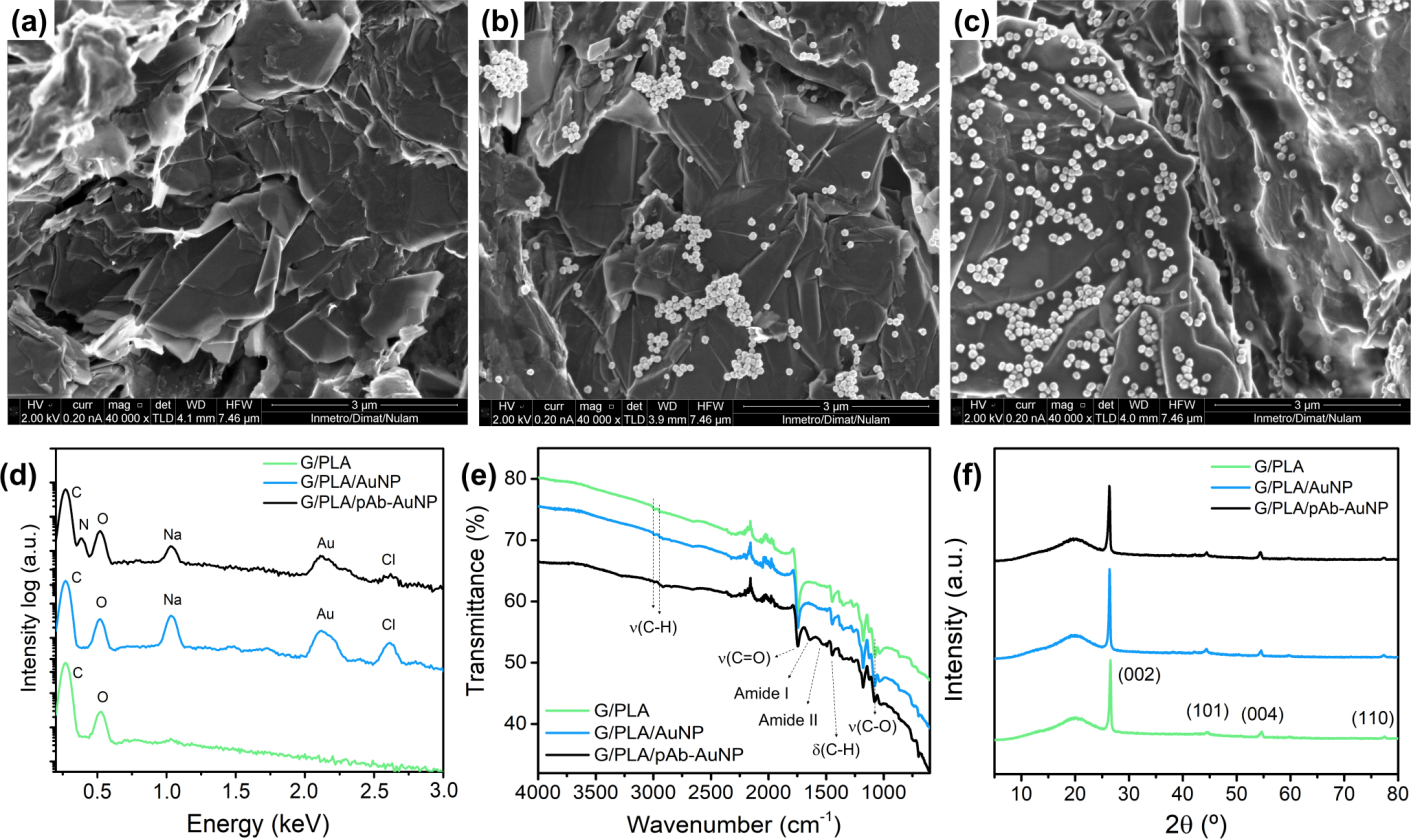
SEM images of (a) G/PLA, (b) G/PLA/AuNP, and (c) G/PLA/pAb–AuNP electrodes at same magnification, (d) EDS spectra, (e) ATR-FTIR spectra and (f) XRD pattern of G/PLA (green), G/PLA/AuNP (blue), and G/PLA/pAb–AuNP (black).

EDS spectra (Figure 2d) and mapping (Figures S3–S5) were performed to further characterize the surface of the electrodes and the changes upon functionalization. For the EDS spectra (Figure 2d), the unmodified G/PLA electrode exhibited only C and O signals, due to the graphite and the polymeric matrix of the electrode, as expected. After the AuNP immobilization (G/PLA/AuNP), characteristics Au peaks were observed, confirming the successful incorporation of the AuNPs. Also, Na and Cl peaks appeared because of the sodium citrate coating the AuNP as well as residual chlorine from the gold salt precursor used for the AuNP synthesis. For the electrode functionalized with the bioconjugates (G/PLA/pAb–AuNP), the main difference observed was the appearance of an additional peak at 0.392 keV, corresponding to the N from amine and amide groups in the antibody structure. The EDS mapping (Figures S3–S5) indicated that the elements were homogeneously distributed across the surface of the electrodes, which indicates a good surface modification for both AuNP and pAb–AuNP functionalization through drop-casting method.

The infrared spectra of the electrodes (G/PLA, G/PLA/AuNP, and the G/PLA/pAb–AuNP) are shown in Figure 2e. As expected, since PLA is the predominant component of the electrode, all characteristic absorption bands of the polymer are observed. The signals at approximately 2992 and 2942 cm^–1^ correspond to C–H stretching vibrations, while the band at 1445 cm^–1^ arises from C–H bending.^31,32^ A strong absorption at 1745 cm^–1^ is assigned to the ester C=O stretching vibration,^33,34^ and several bands in the 1180–1035 cm^–1^ region are attributed to C–O stretching of the ester groups.^31,32^

The successful immobilization of antibodies on the electrode surface (G/PLA/pAb–AuNP) is evidenced by the presence of characteristic amide I and amide II bands at 1637 and 1520 cm^–1^, respectively. The amide I band, sensitive to protein secondary structure, is mainly associated with C=O stretching with minor N–H bending contributions, whereas the amide II band arises from N–H bending coupled with C–N stretching.^35,36^

X-ray diffraction measurements were carried out to evaluate possible structural changes in each stage of electrode modification. X-ray diffraction patterns of all three electrodes (G/PLA, G/PLA/AuNP, and the G/PLA/pAb–AuNP) showed broad peaks around 2θ = 20°, which are characteristic of the PLA polymer.^37^ Moreover, a sharp peak at 2θ = 26.5°, together with additional peaks of lower intensity at 2θ = 44.5°, 54.5°, and 77.5°, can be assigned to the characteristic diffraction pattern of graphitic materials, corresponding to the (002), (101), (004), and (110) planes, respectively.^38–41^ Notably, there were no significant changes or shifts detected after the successive surface modifications, since these steps do not alter the bulk crystallinity of the material. Additionally, the characteristic pattern of AuNPs^42^ were not observed in the diffractograms of G/PLA/AuNP and G/PLA/pAb–AuNP, most likely due to their nanoscale dimensions and low concentration compared to the G/PLA matrix.

Contact angle (θ) measurements were performed to evaluate surface wettability changes after each modification step. The contact angles obtained for G/PLA, G/PLA/AuNP, and G/PLA/pAb–AuNP were 71°, 63°, and 62°, respectively (Figure S6). All measured angles fell between 10° and 90°, indicating a hydrophilic surface.^43^ However, it is important to notice that the surface modification with AuNP and pAb–AuNP provoked a decrease in the θ values, indicating an improved hydrophilicity.

### Electrochemical Characterization

For the biosensor preparation, the pAb–AuNP bioconjugate was immobilized onto the G/PLA electrode surface by drop-casting. Figure 3a shows the cyclic voltammetry (CV) curves of the bare G/PLA electrode (green) and after functionalization with pAb–AuNPs (black). The G/PLA electrode displayed anodic and cathodic peaks characteristic of the [Fe­(CN)_6_]^3–/4–^ redox couple. After functionalization, the CV of the biosensor (G/PLA/pAb–AuNP) exhibited a significant decrease in both anodic and cathodic peak currents. This reduction is most likely related to the presence of antibody-coated AuNPs forming a nonconductive layer on the electrode surface, which hinders electron transfer and thus limits the interaction of the redox probe with the electrode.^44–46^

**Figure 3:**
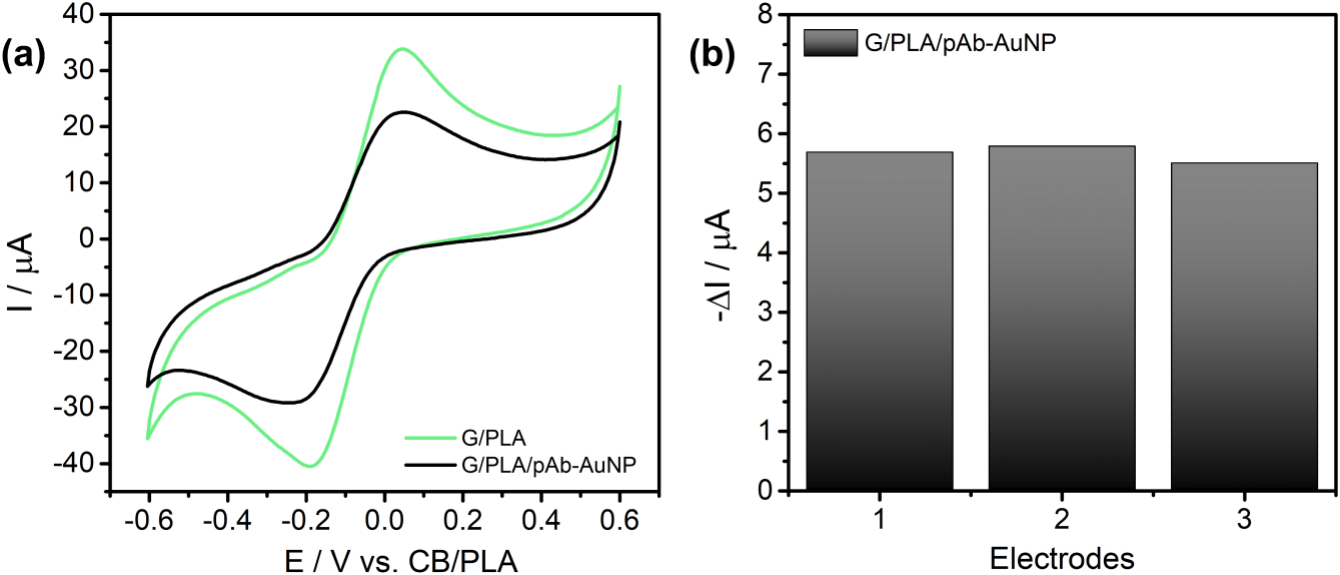
(a) Cyclic voltammetry of the unmodified electrode G/PLA (green) and the biosensor G/PLA/pAb–AuNP (black); (b) reproducibility assay for the preparation of the G/PLA/pAb–AuNP biosensor. Cathodic peak current variation for three independently G/PLA/pAb–AuNP biosensors.

To evaluate the reproducibility of the drop-casting in biosensor fabrication, three electrodes were independently prepared under identical conditions. As shown in Figure 3b, the reduction peak current was 5.66 ± 0.14 μA, corresponding to a relative standard deviation (RSD) of 2.5%. The low RSD and consistent current values demonstrate the high reproducibility of the surface modification process.^47,48^

### Interaction Time

To determine the optimal interaction time between the G/PLA/pAb–AuNP biosensor and the S Ptn, a time-dependent assay was carried out. A 5 μg L^–1^ S Ptn (400 μL) dispersion was applied onto the 3-in-1 3D-printed electrode set (working, counter, and reference electrodes), and different incubation times (1, 5, 10, 15, 20, 25, and 30 min) were tested. The CV responses (Figure 4a) showed a progressive decrease in the cathodic peak current with increasing incubation time, consistent with the gradual binding of the S Ptn to the functionalized working electrode (G/PLA/pAb–AuNP). This binding blocks the electrode surface, reducing the accessibility of the redox probe and thereby lowering the electrochemical signal. Importantly, a 5 min incubation was sufficient to produce a clear signal change, making longer incubation times unnecessary. Thus, 5 min was chosen for subsequent experiments, aligning with the development of a rapid electrochemical biosensor.

**Figure 4:**
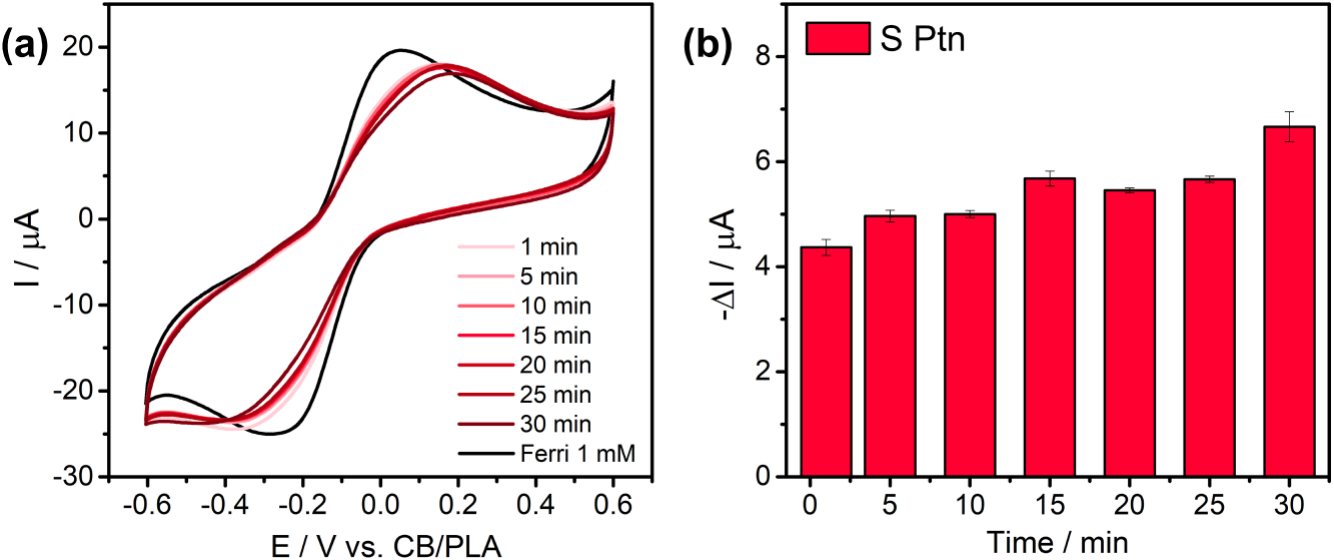
Time-dependent immunoassay ranging from 1 to 30 min of interaction time with S Ptn (a) voltammograms and (b) current variation of the cathodic peak.

### S Ptn Detection

Reproducibility of the G/PLA/pAb–AuNP biosensor toward the S Ptn was evaluated by fabricating three independent electrodes and performing the detection assays under identical conditions (Figure 5a and b). At 5 μg L^–1^ S Ptn, CV measurements showed a consistent decrease in the cathodic peak current of 5.55 ± 0.16 μA (RSD 2.9%), demonstrating the reproducibility required for practical use.

**Figure 5:**
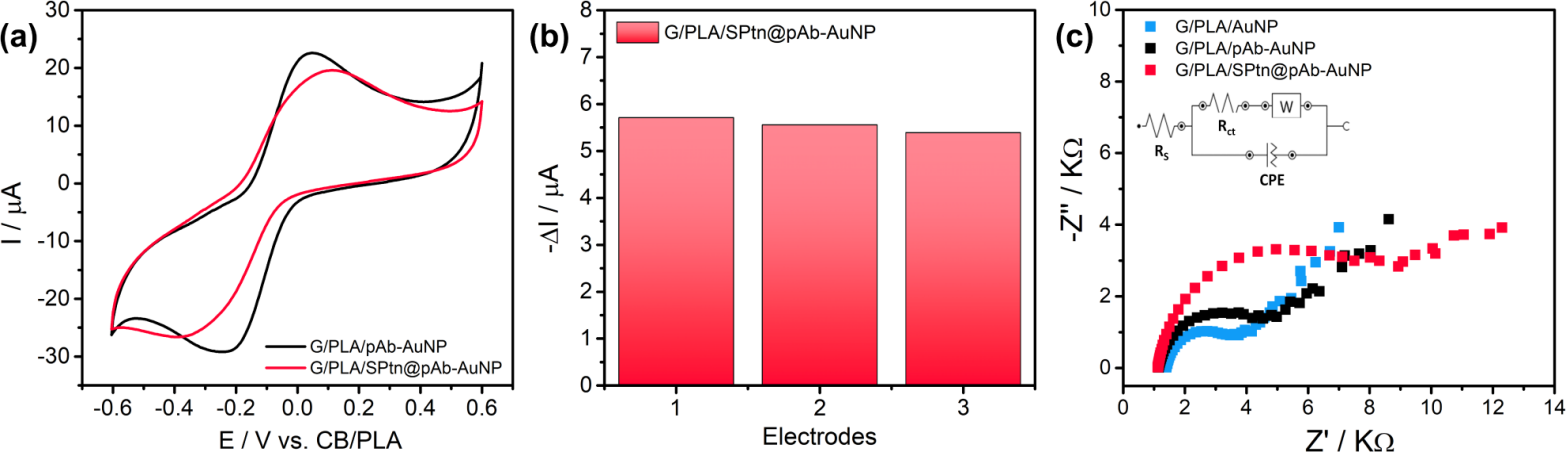
S Ptn recognition. (a) Reproducibility assay at 5 μg L^–1^; (b) corresponding changes in cathodic peak current; (c) EIS Nyquist plots for G/PLA/AuNP (blue), G/PLA/pAb–AuNP (black), and G/PLA/SPtn@pAb–AuNP (red). Inset: Randles equivalent circuit. EIS conditions: 1 kHz–10 mHz, 10 points per decade, 10 mV AC amplitude, at a half-wave potential of −0.060 V (vs CB/PLA electrode).

Electrochemical impedance spectroscopy (EIS) with the [Fe­(CN)_6_]^3–/4–^ redox couple was used to probe changes at the electrode/dispersion interface during S Ptn binding. The Nyquist plots in Figure 5c show a progressive increase in semicircle diameter from G/PLA/AuNP to G/PLA/pAb–AuNP and, after exposure to S Ptn to G/PLA/pAb–AuNP (G/PLA/SPtn@pAb–AuNP), indicating the sequential incorporation of biomolecules (antibody and S Ptn) onto the 3D-printed electrodes increases the charge-transfer resistance (*R*_ct_). The fitted *R*_ct_ values were 2.1, 3.6, and 7.5 kΩ for G/PLA/AuNP (blue), G/PLA/pAb–AuNP (black), and G/PLA/SPtn@pAb–AuNP (red), respectively. These results are consistent with the CV data, in which S Ptn recognition blocks the electrode surface, decreases the faradaic current, and concomitantly increases *R*_ct_.

The heterogeneous electron transfer rate constant (*k*_0_) was determined from EIS data based on R_ct_ values reported in the literature,^49,50^ for the studied electrode surfaces using the [Fe­(CN)_6_]^3–/4–^ redox couple. The calculation was performed according to eq 1:

1 $$R_{\mathrm{ct}}=\frac{RT}{F^{2}k_{0}C}$$

where *R*_ct_ represents the charge transfer resistance obtained from EIS data, *R* is the gas constant, *T* is the absolute temperature, *F* is the Faraday constant, and *C* is the concentration of the electroactive species (1.0 mmol L^–1^). The surface containing only AuNPs exhibited a more pronounced kinetic behavior (*k*_0_ = 6.30 × 10^–4^ cm s^–1^) compared to the biofunctionalized surface G/PLA/pAb–AuNP (*k*_0_ = 1.81 × 10^–4^ cm s^–1^). This indicates that the electron transfer reactions could occur approximately 3.5 times faster on the electrode modified solely with AuNPs. The reduction in *k*_0_ observed at the biosensor surface can be attributed to the partial blocking effect of the antibody immobilization layer, which introduces an additional barrier to charge transfer between the redox probe and the electrode. This behavior is consistent with the EIS results discussed below, confirming the successful attachment of the biorecognition element and its influence on the biosensor’s electrochemical kinetics.

### Biosensor Sensitivity Evaluation

The biosensor G/PLA/pAb–AuNP sensitivity (immunoassays) was evaluated using different concentrations of S Ptn (*C*_SPtn_) ranging from 2.5 to 20 μg L^–1^, with a 5 min incubation time before each measurement (Figure 6). At low S Ptn concentration, the voltammograms exhibited a concentration-dependent decrease in the cathodic peak current, and a linear response was obtained in the range from 2.5 to 10 μg L^–1^. This result demonstrated the biosensor’s potential for quantitative detection of S Ptn, using the calculated analytical curve −Δ*I* (μA) = 0.67 × *C*_SPtn_ (μg L^–1^) + 2.14 (*R* ^2^ = 0.9993), Figure 6c.

**Figure 6:**
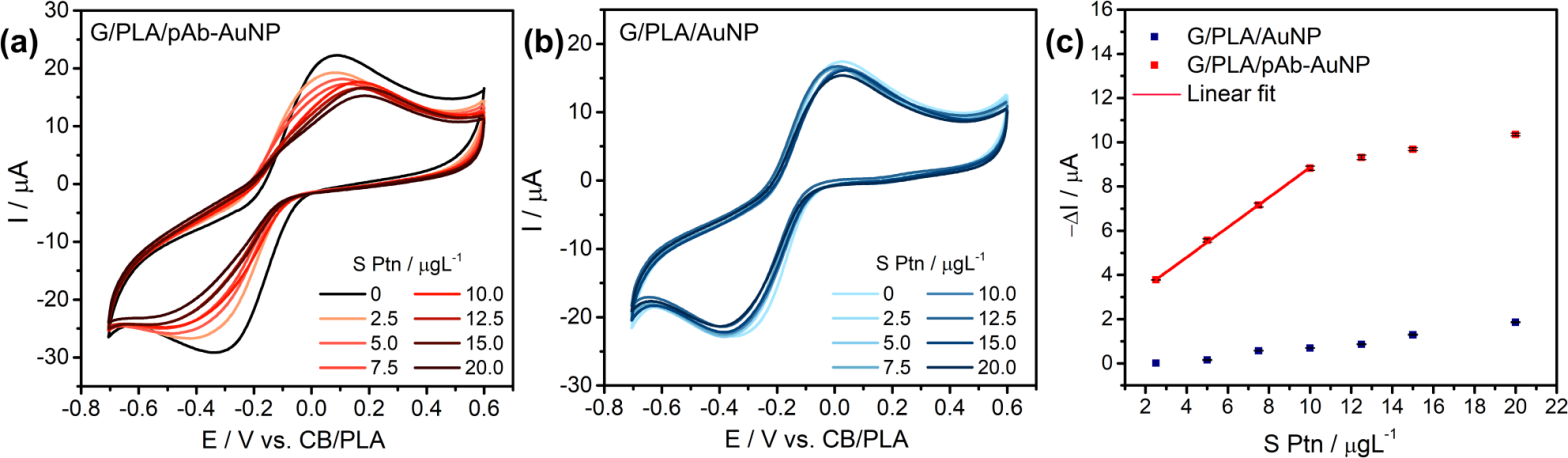
Immunoassay response versus S Ptn concentration. (a) CVs for G/PLA/pAb–AuNP; (b) CVs for G/PLA/AuNP; (c) calibration curves for G/PLA/pAb–AuNP (red) and G/PLA/AuNP (blue).

The calibration data (Figure 6c) were used to estimate the limit of detection (LOD) and limit of quantification (LOQ) according to LOD = (3.3 × σ)/*b*, and LOQ = 3 × LOD, where σ is the standard deviation of the calibration-curve intercept and *b* is the slope (sensitivity).^26,51^ The resulting values were LOD = 0.33 μg L^–1^ (0.76 pM) and LOQ = 0.99 μg L^–1^ (2.28 pM). Increasing S Ptn concentration (red curve in Figure 6c) increases surface coverage via binding to antibody paratopes on the electrode. When these sites are fully occupied (site saturation), additional protein contributes negligibly to interfacial blocking, and the voltammetric current approaches a plateau.

To investigate the contribution of the antibody and assess nonspecific adsorption, the same concentration-dependent assay was performed with an electrode functionalized only with AuNPs (no antibody), G/PLA/AuNP. As shown in Figure 6b, adding S Ptn produced only a slight decrease in the cathodic peak current, consistent with minimal nonspecific adsorption (e.g., electrostatic or van der Waals interactions).^52^ These observations indicate that the stronger signal change seen with G/PLA/pAb–AuNP requires antibody conjugation to the AuNPs, and is attributable to antibody–antigen binding.

### Specificity Tests

To evaluate analytical specificity, the G/PLA/pAb–AuNP biosensor was challenged with proteins from other viruses circulating in Brazil. CV measurements were performed using 10 pM solutions of CHKV E2 protein and the NS1 proteins from ZIKV and DENV1 (Figure S7). The resulting current changes (Δ*I*) were compared with those obtained for the SARS-CoV-2 S Ptn under identical conditions (Figure 7).

**Figure 7:**
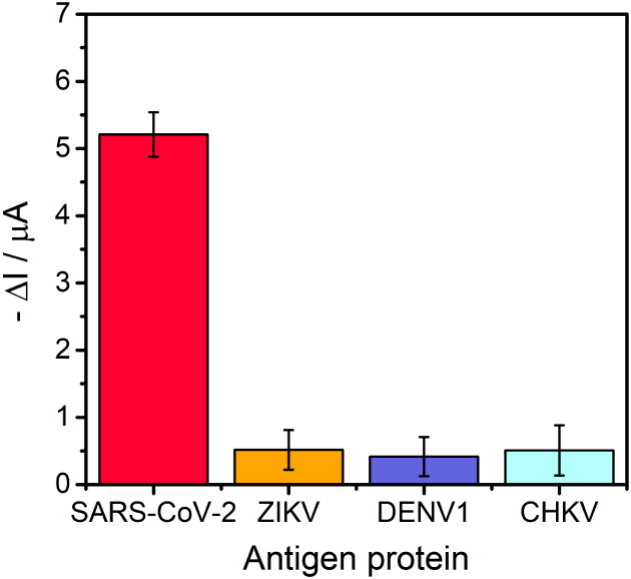
Electrochemical immunoassay responses to 10 pM antigen dispersions. Bar charts correspond to SARS-CoV-2 S Ptn (red), ZIKV (yellow) NS1 Ptn, DENV1 (purple) NS1 Ptn, and CHKV E2 Ptn (blue).

As shown in Figure 7, the −Δ*I* for the SARS-CoV-2 S Ptn is markedly larger than the signals recorded for the other antigens. The minimal current changes for CHKV E2, ZIKV NS1, and DENV1 NS1 indicate negligible cross-reactivity, confirming biosensor’s high specificity.

### Applicability in Human Serum

To assess applicability in a complex matrix, the standard addition method was applied to human serum spiked with S Ptn at two concentrations (2.5 and 3.5 μg L^–1^; Figure 8). The measured concentrations were 2.43 and 3.52 μg L^–1^, yielding recoveries of 97.2% and 100.7%, respectively. These values are very close to the nominal concentrations, with small, opposite-signed biases (−2.8% at 2.5 μg L^–1^ and +0.7% at 3.5 μg L^–1^). The near-quantitative recoveries from serum constituents are minimal within this range. In practical terms, the data support good accuracy for the assay in a complex biological matrix, reinforcing the suitability of the G/PLA/pAb–AuNP biosensor for quantitative measurements in real samples.^53^

**Figure 8:**
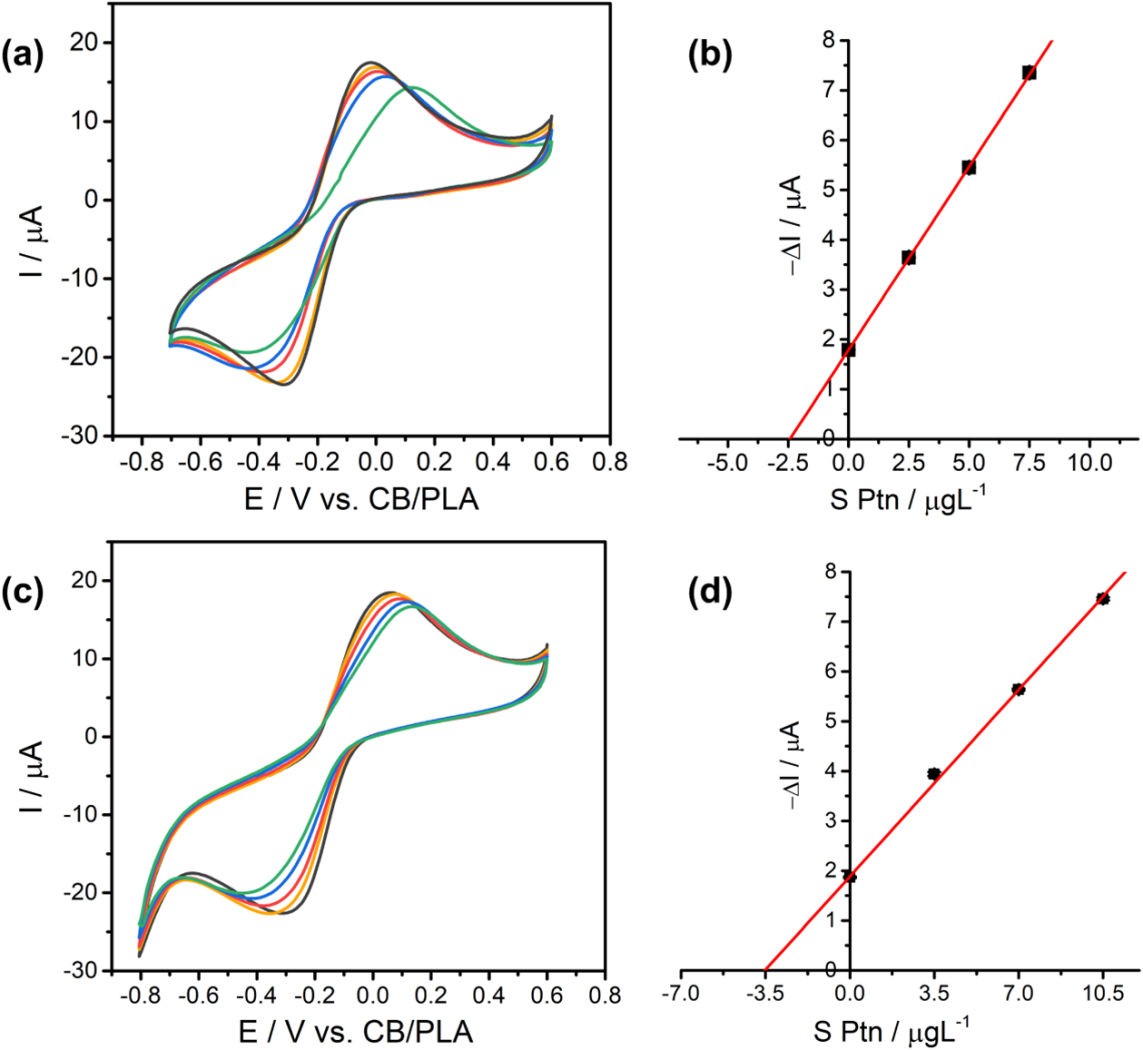
Standard addition method for S Ptn detection in human serum using the G/PLA/pAb–AuNP biosensor. (a) CV at 2.5 μg L^–1^ concentration; (b) the corresponding calibration curve; (c) CV at 3.5 μg L^–1^ concentration; (d) the corresponding calibration.

To evaluate the G/PLA/pAb–AuNP biosensor performance, a comparison with other CV-based biosensors reported in the literature for SARS-CoV-2 diagnosis is displayed in Table 1. The comparison considers the type of bioreceptor, target analyte, linear detection range, the LOD, and the interaction time. Overall, the target analytes vary across studies, from the SARS-CoV-2 biomarkers, such as antibodies, genome sequences, or viral proteins, to the whole virus particle. This change is accompanied by the different bioreceptors used.

**Table 1:** Comparative Performance of Electrochemical Biosensors Using Cyclic Voltammetry for SARS-CoV-2 Diagnosis Reported in the Literature

Analyte	Bioreceptor	Linear range (pM)	LOD (pM)	Interaction time (min)	Ref.
SARS-CoV-2 antibody	S Ptn	500–3500	270	10	^55^
SARS-CoV-2 antibody	S Ptn	1–90 × 10^3^	334	40	^56^
SARS-CoV-2 cDNA	SARS-CoV-2 cDNA-SH	1 × 10^6^ to 50.0 × 10^6^	3 × 10^5^	30	^25^
S Ptn	Concanavalin A	-	456^a^	30	^57^
S Ptn	Anti-S Ptn antibody	10–4500	2.7	60	^24^
S Ptn	Peptide	0.165–1.65 × 10^5^ ^a^	0.132^a^	20	^49^
S Ptn	Anti-S Ptn antibody	5.7 – 22.8^a^	0.76^a^	5	This work

a Calculated using the molecular mass of S Ptn 438.26 kDa;^58^ cDNA: SARS-CoV-2 virus complementary DNA strand (target sequence); cDNA-SH: thiol-modified SARS-CoV-2 virus complementary DNA strand (probe sequence).

Most CV-based electrochemical platforms require 20–60 min of incubation to achieve sufficient analyte–bioreceptor binding (Table 1), which is relatively long for rapid testing. By contrast, the G/PLA/pAb–AuNP biosensor developed here achieved a good response with LOD of 0.76 pM after only 5 min of interaction—at least a 4-fold shorter interaction time—representing a significant advance in assay speed.

Therefore, our platform offers competitive sensitivity while maintaining operational simplicity, using a single drop-casting step directly on the surface of 3D-printed electrodes. For instance, the peptide-based biosensor of Kumar et al.^54^ reports slightly lower LOD but requires 20 min incubation and involves multiple manual fabrication steps, factors that may limit scalability and reproducibility. In addition, our device maintained near-quantitative recoveries in human serum (97–101%), indicating tolerance to complex matrices, and exhibited low between-electrode RSD (≈2.5–2.9%), supporting practical robustness. Although preparing the antibody-bound AuNP conjugate involves three steps, it can be made in batches, stays colloidally stable,^4,6,30^ and only ∼20 μL is needed per electrode. This keeps the per-test process simple.

## Conclusions

In this work, we fabricated 3-in-1 3D-printed electrodes and modified them with spherical AuNPs conjugated to polyclonal antibodies against the SARS-CoV-2 S Ptn. The resulting G/PLA/pAb–AuNP biosensor enabled indirect detection by cyclic voltammetry, with a limit of detection of 0.76 pM and a linear range of 2.5–10 μg L^–1^. Binding of the antigen to the surface-immobilized antibodies blocked the electrode and reduced the cathodic peak current. The assay time was 5 min, which is shorter than most CV reported systems (20–60 min). The device showed good reproducibility (RSD ≈ 2.5–2.9%) and maintained performance in human serum, with recoveries of 97–101%, indicating minimal matrix effects. Control electrodes without antibody produced only small signal changes, and tests with nontarget viral proteins (CHKV E2, ZIKV NS1, DENV-1 NS1) gave near-baseline responses, showing that the signal arises from specific antibody–antigen recognition. Therefore, the short assay time, low LOD, and accurate performance in serum indicate that this platform advances 3D-printed electrochemical biosensors toward practical, cost-effective point-of-care detection of SARS-CoV-2.

## Supplementary Material





## Notes

The 3D-printed electrode .stl files (ZIP)
SEM images and EDS mapping of the electrodes; voltammetry measurements of the specificity assay and technical drawing of the 3D-printed electrode (PDF)
