# Supplementary material for: Rapid Detection of SARS-CoV‑2 Spike Protein Using a Fully 3D-Printed Electrochemical Biosensor
Source: ACS Omega. 2025 Dec 11;10(50):62025–35. doi: 10.1021/acsomega.5c09067 (PMC12750377; doi:10.1021/acsomega.5c09067)
Supplement: Supplementary file 2 [file ao5c09067_si_002.pdf]

SUPPORT INFORMATION

Rapid Detection of SARS-CoV-2 Spike Protein Using a Fully 3D-Printed Electrochemical Biosensor

Dayenny L. D'Amato,[a] *Natália M. Caldas,*[b] *Lucas V. de Faria,*[c] *Ana Beatriz C. Souza,*[a] *Guilherme P. Oliveira,*[b] *Diego Costa,*[a] *Mikaelly O. B. de Sousa,*[a] *Rafael M. Dornellas,**[b] *Célia M. Ronconi**[a]

[a]Departamento de Química Inorgânica, Universidade Federal Fluminense, Campus do Valonguinho, Outeiro São João Batista s/n, Centro, Niterói, RJ, 24020-150, Brazil.

[b]Departamento de Química Analítica, Universidade Federal Fluminense, Campus do Valonguinho, Outeiro São João Batista s/n, Centro, Niterói, RJ, 24020-150, Brazil.

[c]Departamento de Química Analítica, Universidade Federal do Rio de Janeiro, Campus Fundão, Avenida Athos da Silveira Ramos 149, Cidade Universitária, Rio de Janeiro, RJ, 21941-590, Brazil.

Contents

S1. Photograph of the 3D-printed electrochemical sensors

S2. Scanning Electron Microscopy Images

S3. Energy Dispersive Spectroscopy

S4. Contact angle

S5. Specificity tests

S6. Technical Drawing

S1. Photograph of the 3D-printed electrochemical sensor

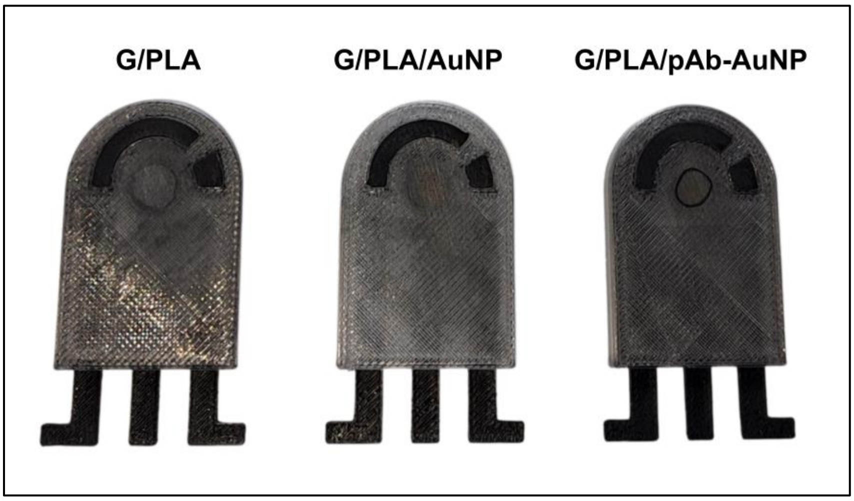

Figure S1. Photographs of the electrodes G/PLA, G/PLA/AuNP, and G/PLA/pAb-AuNP.

S2. Scanning Electron Microscopy (SEM)

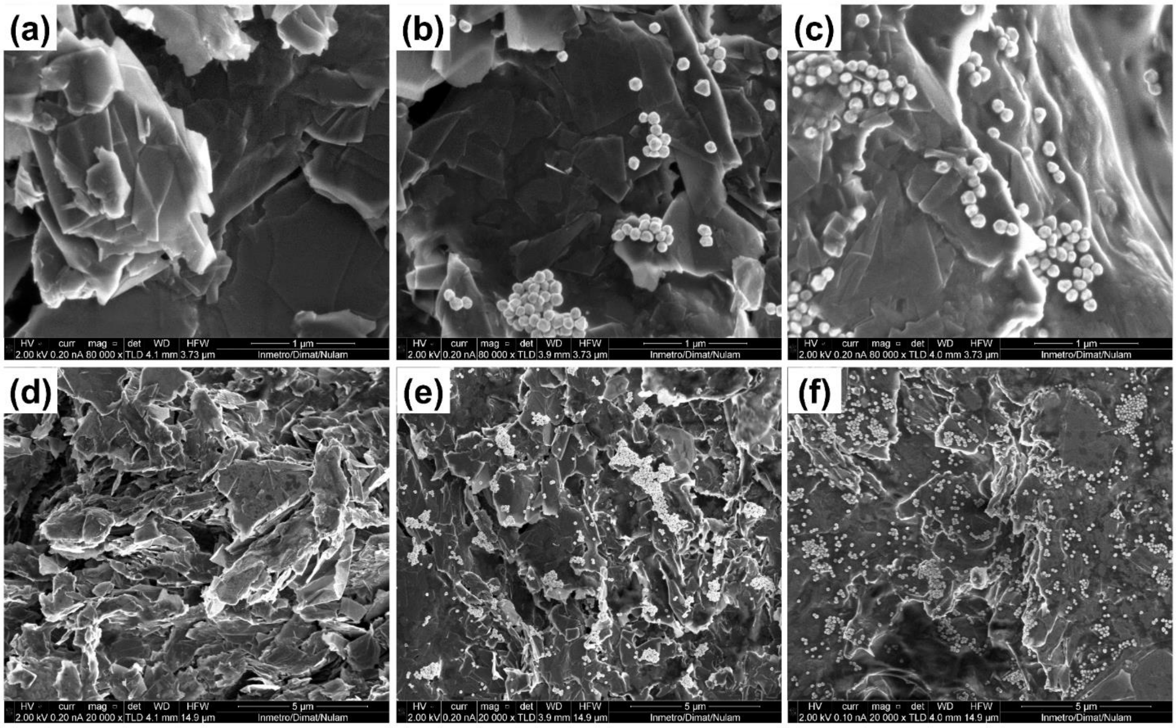

Figure S2. SEM images of the electrodes: **(a)** and **(d)** G/PLA, **(b)** and **(e)** G/PLA/AuNP, **(c)** and **(f)** G/PLA/pAb-AuNP.

S3. Energy Dispersive Spectroscopy (EDS)

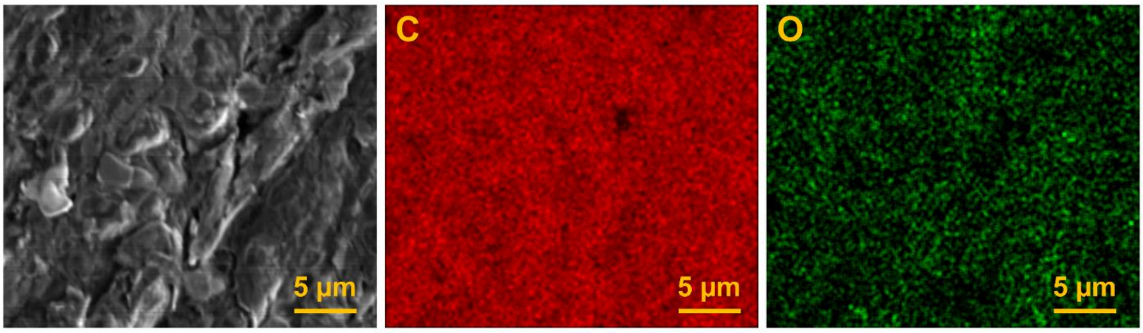

Figure S3. EDS mapping of G/PLA.

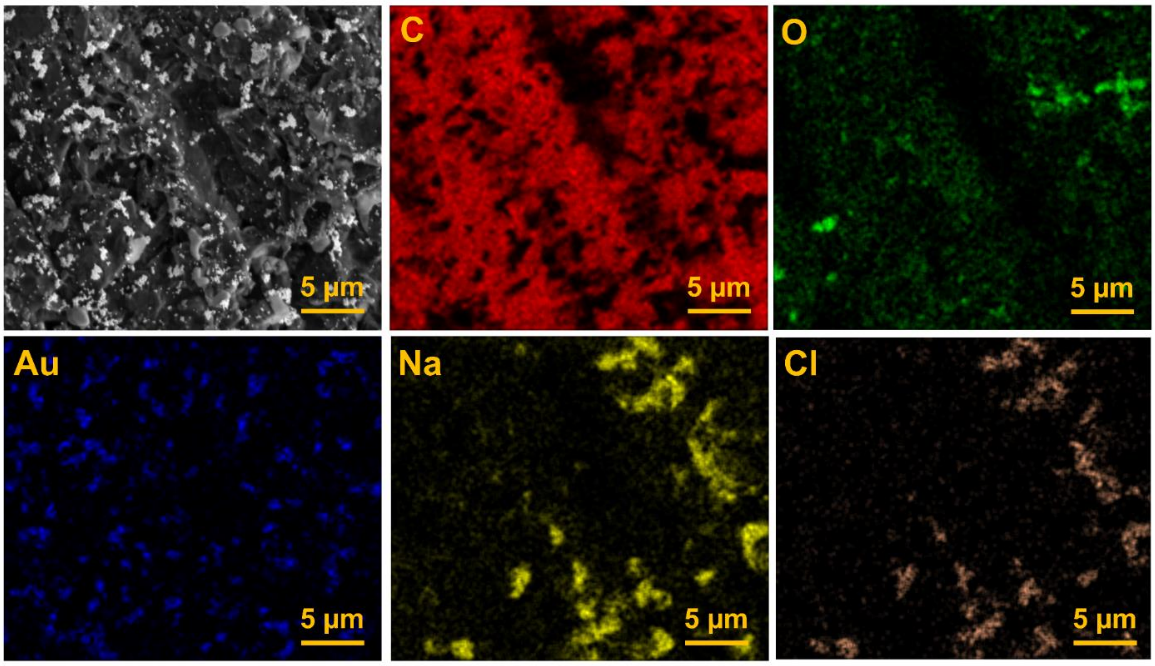

Figure S4. EDS mapping of G/PLA/AuNP.

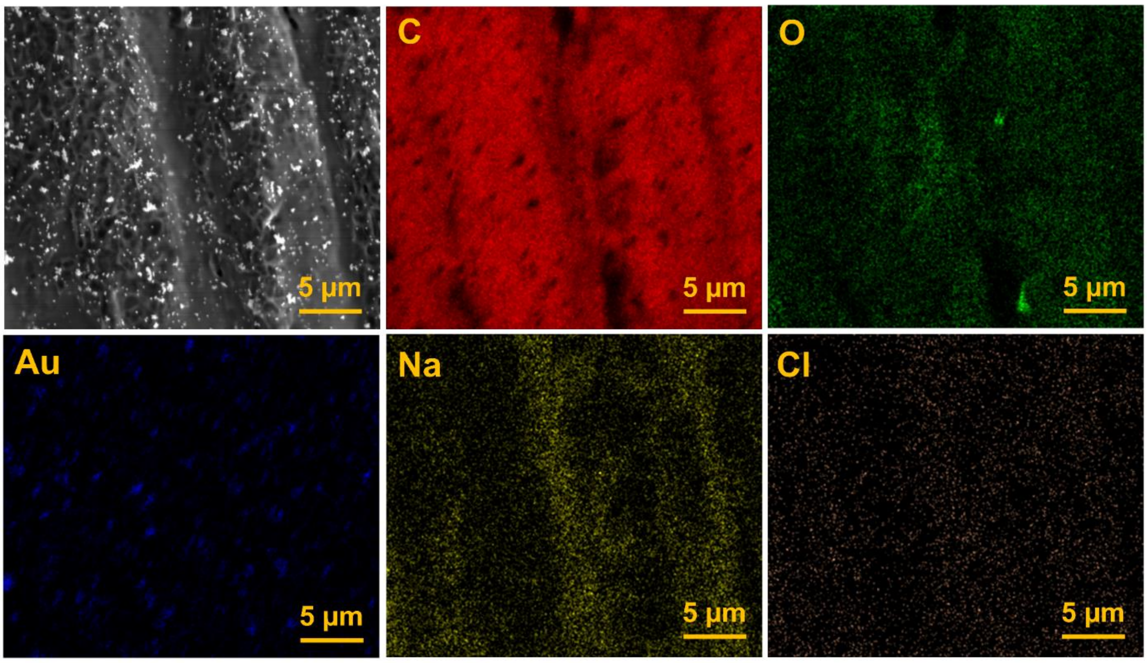

Figure S5. EDS mapping of G/PLA/pAb-AuNP.

S4. Contact angle

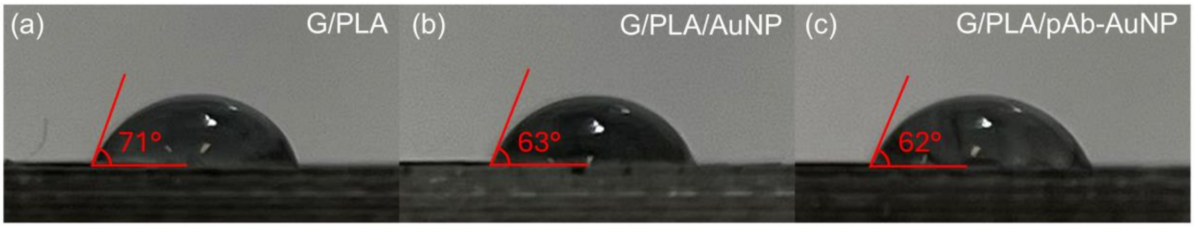

Figure S6. Contact angle measurements of water droplets on the electrodes: **(a)** G/PLA, **(b)** G/PLA/AuNP, **(c)** G/PLA/pAb-AuNP.

S5. Specificity tests

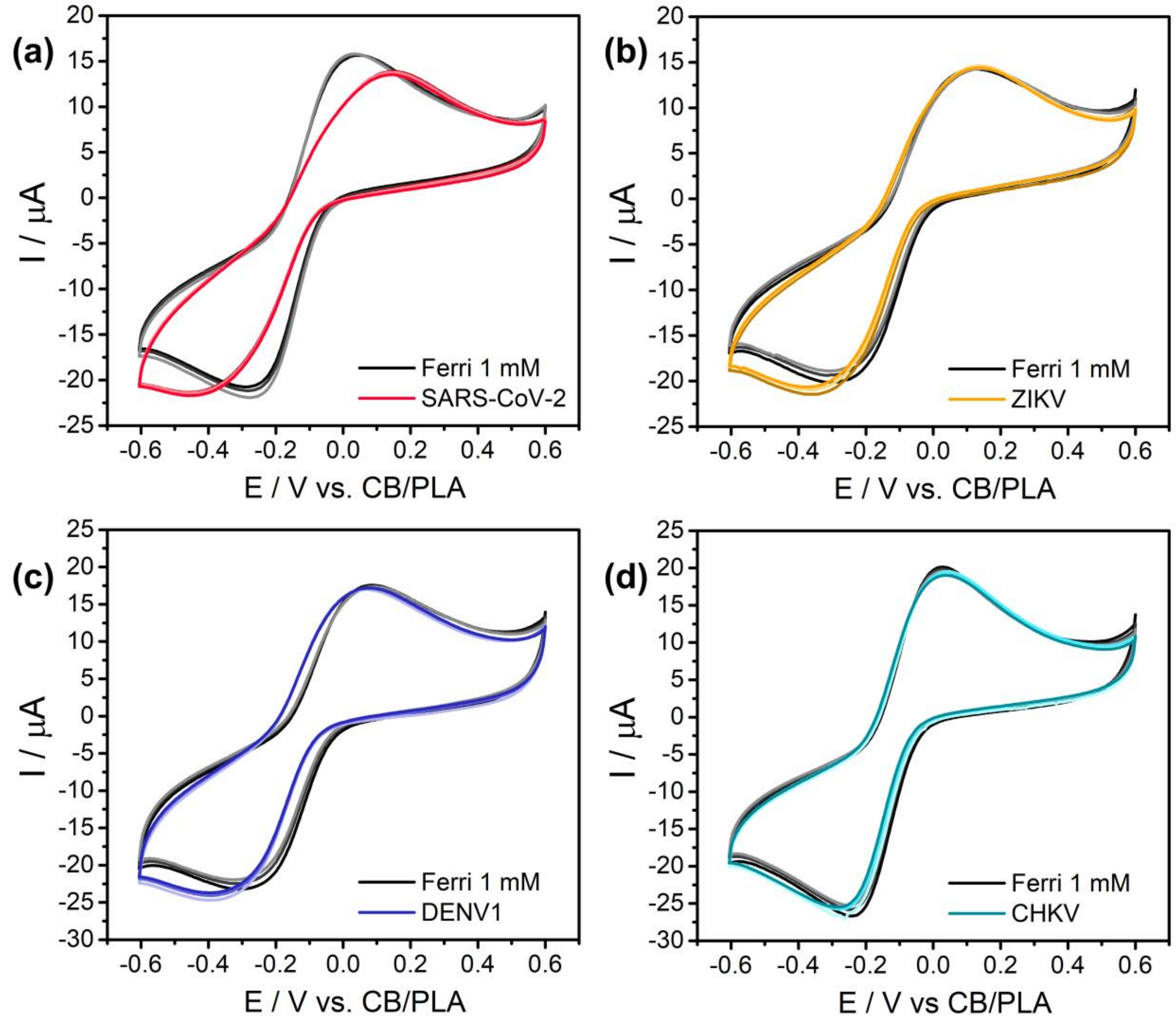

Figure S7. Cyclic voltammetry measurements for the specificity assay with different antigen proteins at a 10 nM concentration **(a)** S Ptn from SARS-CoV-2 (red), NS1 protein from **(b)** ZIKV (yellow) and **(c)** DENV1 (purple), and **(d)** E2 protein from CHKV (blue).

S6. Technical Drawing

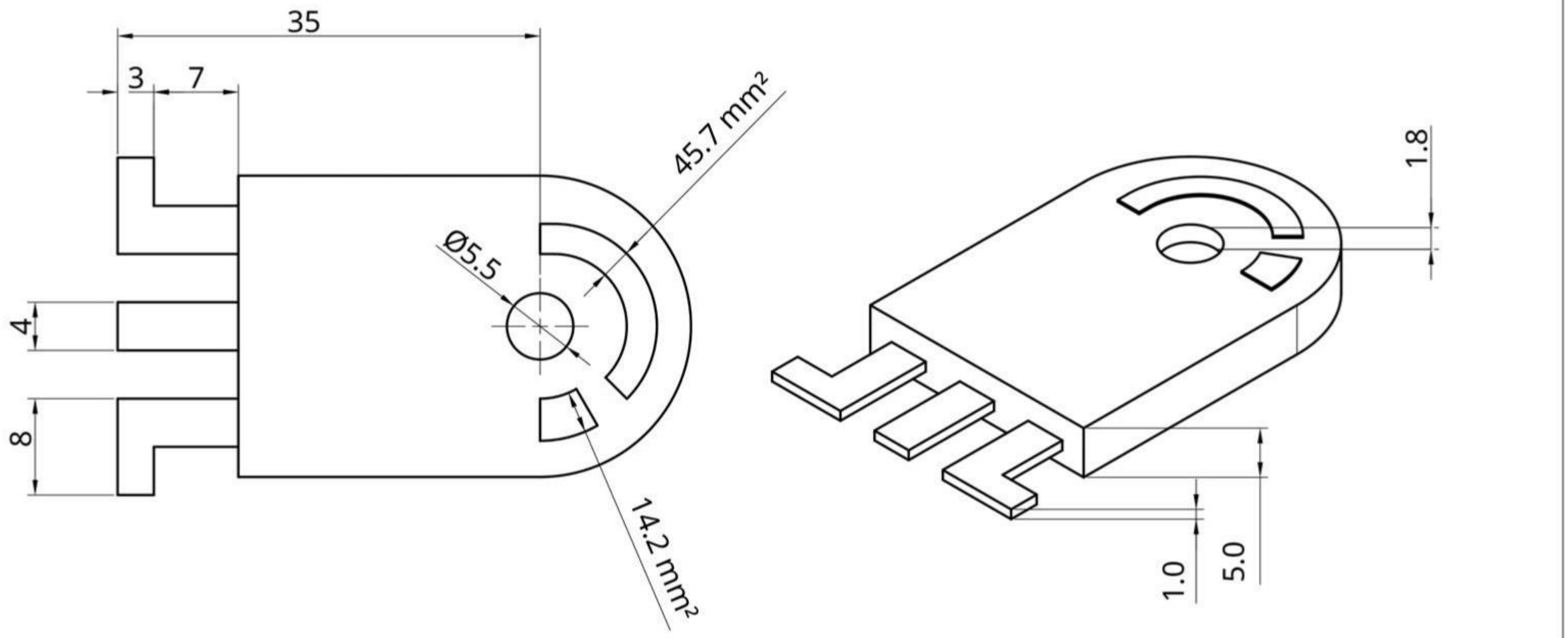

TITLE 3-in-1 3D-printed electrochemical biosensor			
Dayenny L. D'Amato, Natália M. Caldas, Lucas V. de Faria, Ana Beatriz C. Souza, Guilherme P. Oliveira, Diego O. Costa, Mikaelly O. B. de Sousa, Rafael M. Dornellas, Célia M. Ronconi			
SIZE A4		S6	REV. 1
SCALE 2:1	UNIT Millimeters	SHEET 1 of 5	

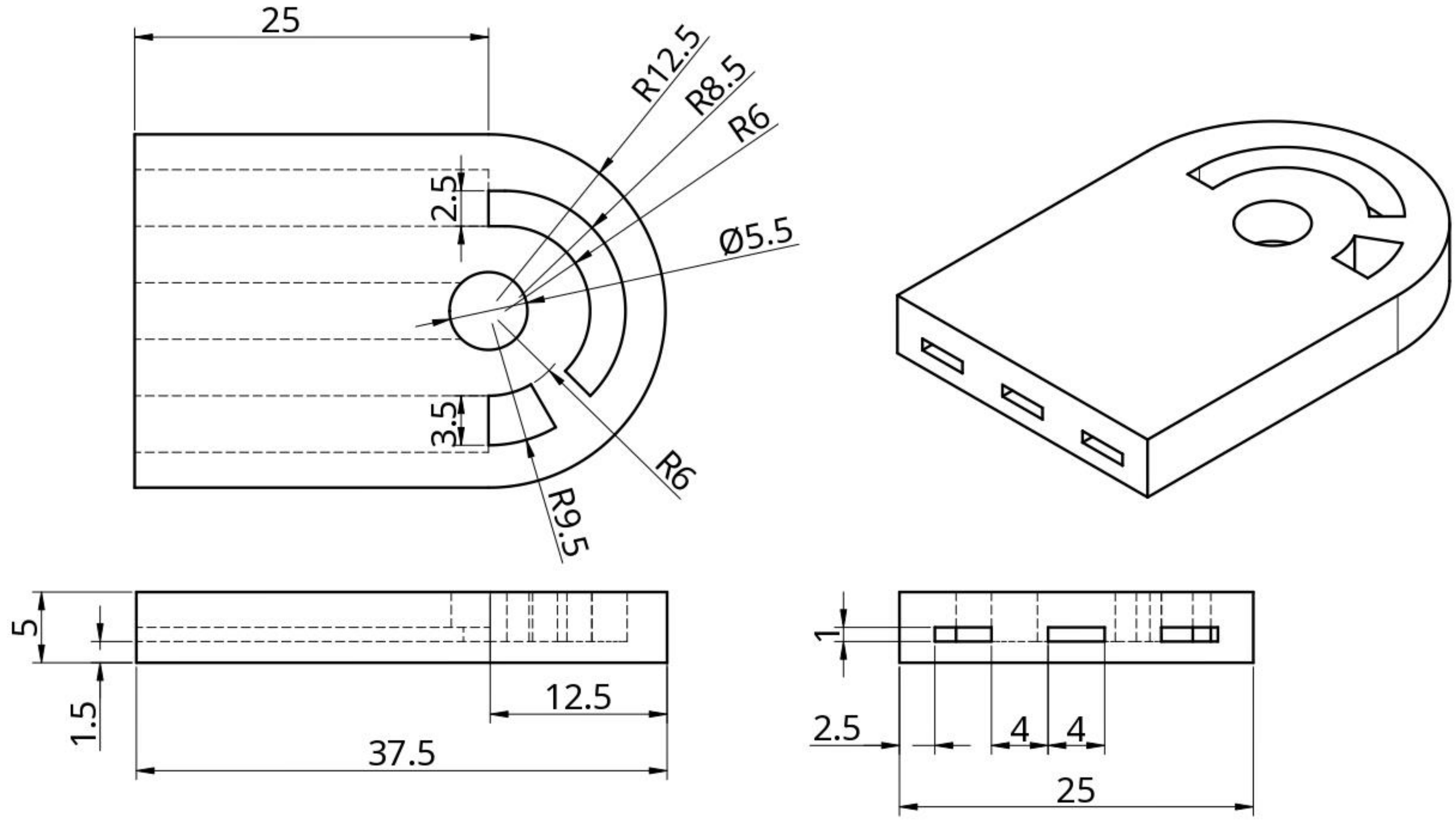

TITLE Non-conductive part			
Dayenny L. D'Amato, Natália M. Caldas, Lucas V. de Faria, Ana Beatriz C. Souza, Guilherme P. Oliveira, Diego O. Costa, Mikaelly O. B. de Sousa, Rafael M. Dornellas, Célia M. Ronconi			
SIZE A4	S7		REV. 1
SCALE 2:1	UNIT Millimeters	SHEET 2 of 5	

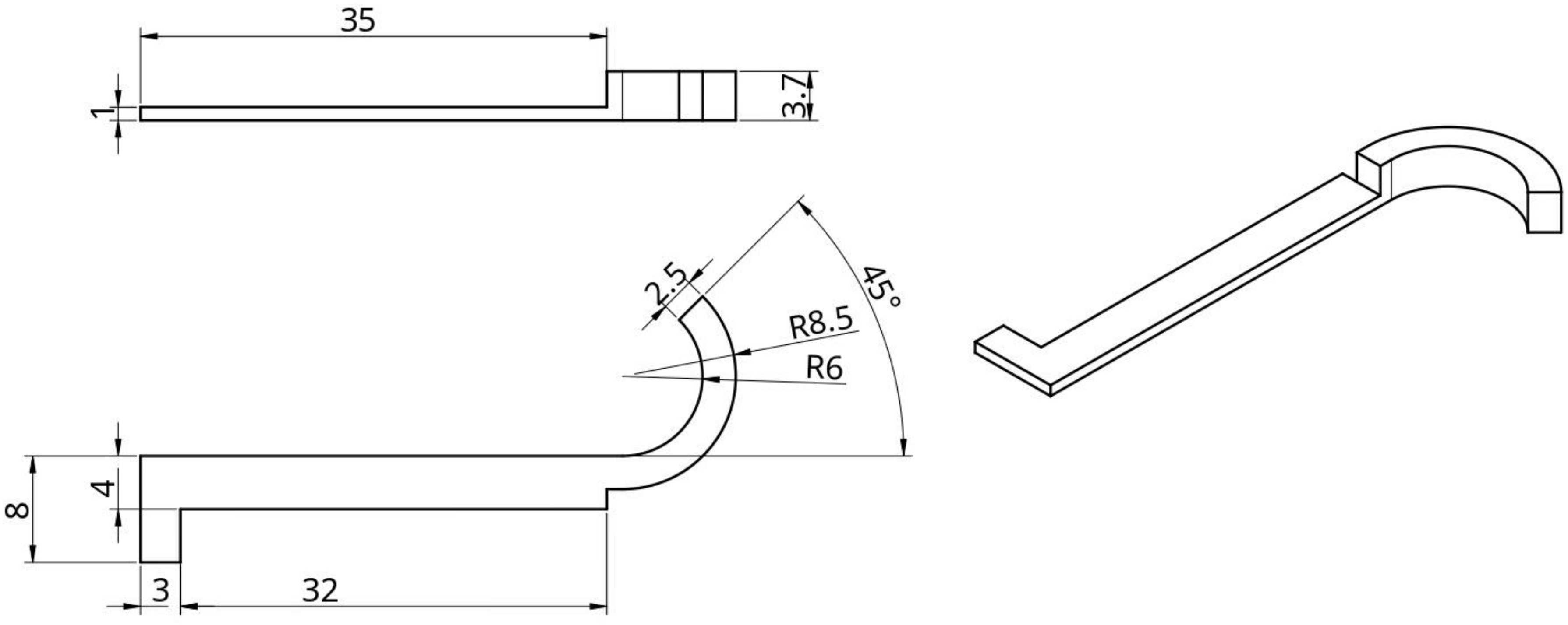

Conductive part: auxiliary electrode

Dayenny L. D'Amato, Natália M. Caldas, Lucas V. de Faria, Ana Beatriz C. Souza, Guilherme P. Oliveira, Diego O. Costa, Mikaelly O. B. de Sousa, Rafael M. Dornellas, Célia M. Ronconi

SIZE A4		S8	REV. 1
SCALE 2:1	UNIT Millimeters	SHEET 3 of 5	

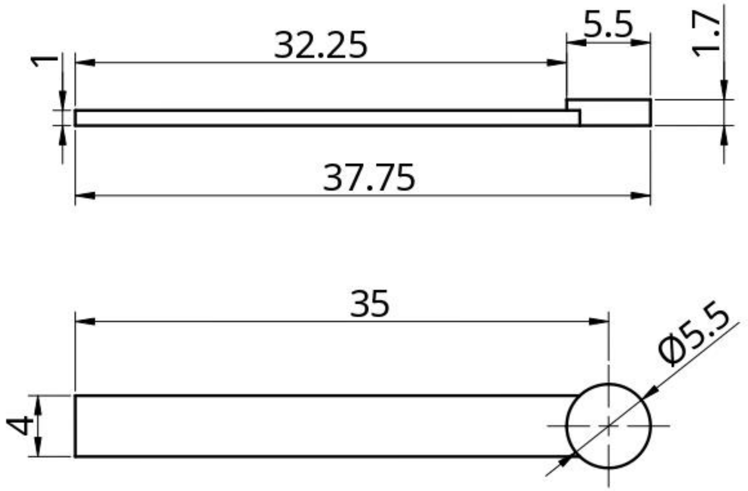

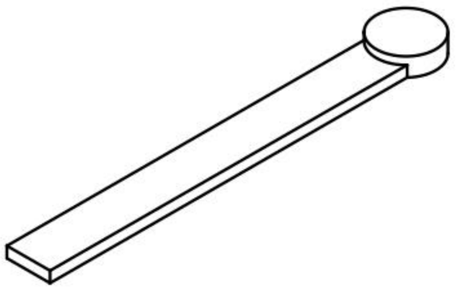

Conductive part: working electrode

Dayenny L. D'Amato, Natália M. Caldas, Lucas V. de Faria, Ana Beatriz C. Souza, Guilherme P. Oliveira, Diego O. Costa, Mikaelly O. B. de Sousa, Rafael M. Dornellas, Célia M. Ronconi

SIZE A4		S9	REV. 1
SCALE 2:1	UNIT Millimeters	SHEET 4 of 5	

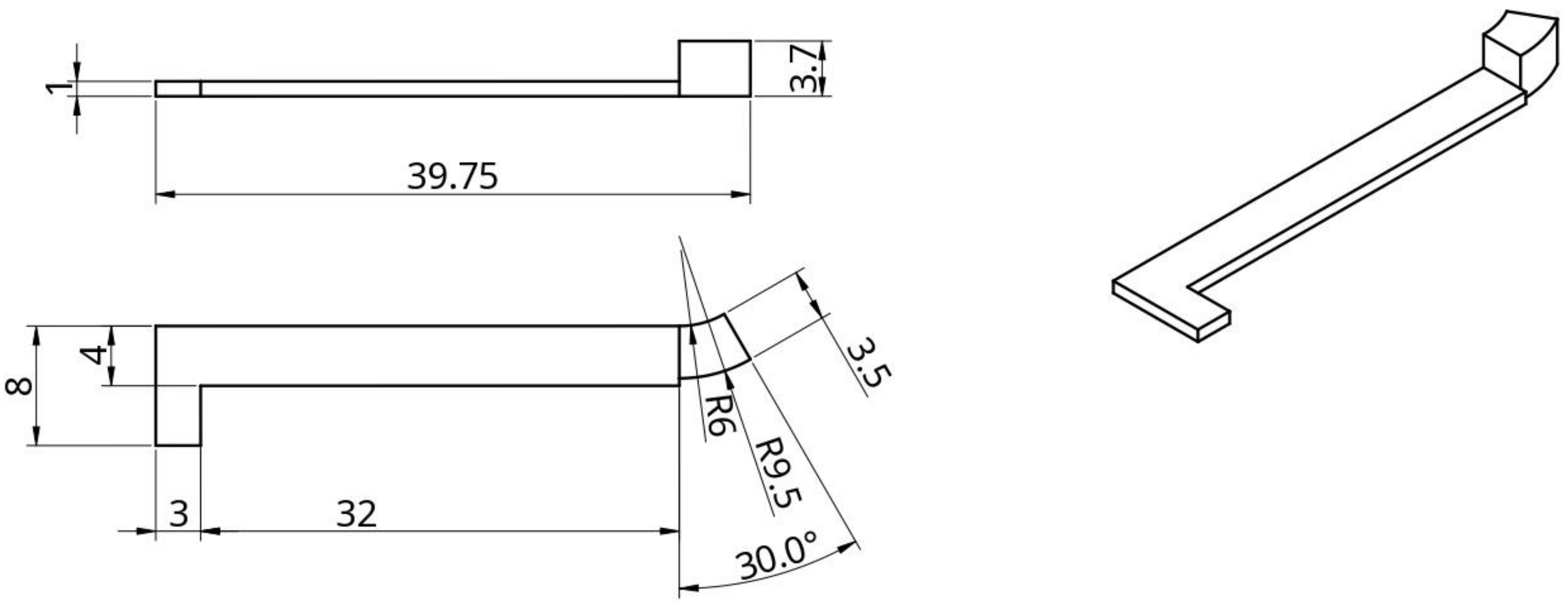

Conductive part: reference electrode

Dayenny L. D'Amato, Natália M. Caldas, Lucas V. de Faria, Ana Beatriz C. Souza, Guilherme P. Oliveira, Diego O. Costa, Mikaelly O. B. de Sousa, Rafael M. Dornellas, Célia M. Ronconi

SIZE A4				S10	REV. 1
SCALE 2:1	UNIT Millimeters		SHEET 5 of 5		
